# Efficacy and safety of carmustine wafers, followed by radiation, temozolomide, and bevacizumab therapy, for newly diagnosed glioblastoma with maximal resection

**DOI:** 10.1007/s10147-024-02650-9

**Published:** 2024-11-11

**Authors:** Masayuki Kanamori, Ichiyo Shibahara, Yoshiteru Shimoda, Yukinori Akiyama, Takaaki Beppu, Shigeo Ohba, Toshiyuki Enomoto, Takahiro Ono, Yuta Mitobe, Mitsuto Hanihara, Yohei Mineharu, Joji Ishida, Kenichiro Asano, Yasuyuki Yoshida, Manabu Natsumeda, Sadahiro Nomura, Tatsuya Abe, Hajime Yonezawa, Ryuichi Katakura, Soichiro Shibui, Toshihiko Kuroiwa, Hiroyoshi Suzuki, Hidehiro Takei, Haruo Matsushita, Ryuta Saito, Yoshiki Arakawa, Yukihiko Sonoda, Yuichi Hirose, Toshihiro Kumabe, Takuhiro Yamaguchi, Hidenori Endo, Teiji Tominaga

**Affiliations:** 1 Department of Neurosurgery, Tohoku University Graduate School of Medicine, Sendai, Japan; 2 Department of Neurosurgery, Kitasato University School of Medicine, Sagamihara, Japan; 3 Department of Neurosurgery, Sapporo Medical University School of Medicine, Sapporo, Japan; 4 Department of Neurosurgery, Iwate Medical University, Shiwa, Japan; 5 Department of Neurosurgery, Fujita Health University, Toyoake, Japan; 6 Department of Neurosurgery, Fukuoka University, Fukuoka, Japan; 7 Department of Neurosurgery, Akita University Graduate School of Medicine, Akita, Japan; 8 Department of Neurosurgery, Faculty of Medicine, Yamagata University, Yamagata, Japan; 9 Department of Neurosurgery, Graduate School of Medicine, University of Yamanashi, Yamanashi, Japan; 10 Department of Neurosurgery, Kyoto University Graduate School of Medicine, Kyoto, Japan; 11 Department of Neurological Surgery, Okayama University Graduate School of Medicine, Dentistry, and Pharmaceutical Sciences, Okayama, Japan; 12 Department of Neurosurgery, Hirosaki University Graduate School of Medicine, Hirosaki, Japan; 13 Department of Neurosurgery, St. Marianna University School of Medicine, Kawasaki, Japan; 14 Department of Neurosurgery, Niigata University Brain Research Institute, Niigata, Japan; 15 Department of Neurosurgery, Yamaguchi University School of Medicine, Ube, Japan; 16 Department of Neurosurgery, Faculty of Medicine, Saga University, Saga, Japan; 17 Department of Neurosurgery, Graduate School of Medical and Dental Sciences, Kagoshima University, Kagoshima, Japan; 18 Department of Neurosurgery, Miyagi Cancer Center, Natori, Japan; 19 Department of Neurosurgery, Teikyo University Hospital, Kawasaki, Japan; 20 Department of Neurosurgery, Osaka Medical and Pharmaceutical University, Takatsuki, Japan; 21 Department of Pathology and Laboratory Medicine, National Hospital Organization Sendai Medical Center, Miyagi, Japan; 22 Department of Pathology and Laboratory Medicine, University of Texas, Houston, USA; 23 Department of Radiation Oncology, Tohoku University Graduate School of Medicine, Sendai, Japan; 24 Department of Neurosurgery, Nagoya University Graduate School of Medicine, Nagoya, Japan; 25 Division of Biostatistics, Tohoku University Graduate School of Medicine, Sendai, Japan

**Keywords:** Glioblastoma, Maximal resection, Temozolomide, Carmustine wafers, Bevacizumab

## Abstract

**Background:**

To improve the outcome in newly diagnosed glioblastoma patients with maximal resection, we aimed to evaluate the efficacy and safety of implantation of carmustine wafers (CWs), radiation concomitant with temozolomide and bevacizumab, and maintenance chemotherapy with six cycles of temozolomide and bevacizumab.

**Method:**

This prospective phase II study enrolled glioblastoma patients considered candidates for complete resection (> 90%) of a contrast-enhanced lesion. The CWs were intraoperatively implanted into the resection cavity after achieving maximal resection. Patients without a measurable contrast-enhanced lesion on magnetic resonance imaging within 48 h after resection received concomitant radiotherapy and chemotherapy with temozolomide and bevacizumab, followed by maintenance treatment with up to six cycles of temozolomide and bevacizumab. The primary endpoint was the 2-year overall survival rate in glioblastoma patients with protocol treatment.

**Results:**

From October 2015 to April 2018, we obtained consent for the first registration from 70 patients across 17 institutions in Japan, and 49 patients were treated according to the protocol. We evaluated the safety in 49 patients who were part of the second registration and the efficacy in 45 glioblastoma patients treated according to the protocol. The profile of hematological and most of the non-hematological adverse effects was similar to that in previous studies, but stroke occurred in 12% of cases (6/49 patients). The estimated 2-year overall survival rate was 51.3%.

**Conclusion:**

Implantation of CWs, followed by concomitant radiation, temozolomide, and bevacizumab, and six cycles of temozolomide and bevacizumab may offer some benefit to survival in Japanese glioblastoma patients with maximal resection.

**Trial ID:**

jRCTs021180007.

**Supplementary Information:**

The online version contains supplementary material available at 10.1007/s10147-024-02650-9.

## Introduction

Glioblastoma is the most malignant primary parenchymal tumor in adults. A randomized phase III trial by the European Organization for Research and Treatment of Cancer (EORTC) and the National Cancer Institute of Canada Clinical Trials Group (NCIC) reported improved median overall survival (OS) for patients with glioblastoma treated with concomitant and adjuvant temozolomide and radiotherapy. This treatment, called Stupp’s regimen, is the standard treatment for newly diagnosed glioblastoma [1]. Even with standard treatment, however, glioblastoma has a poor prognosis with a median OS of 14.6 months and a 2-year survival rate of 26.5% [1].

Extent of resection (EOR) is an important prognostic factor in the treatment of glioblastoma, and several retrospective studies of large numbers of cases report a correlation between the EOR of contrast-enhanced (CE) lesions and prognosis [2, 3]. The 2-year survival rate of combined treatment after complete resection was 38.4% in the EORTC-NCIC clinical trial [1], however, suggesting that more effective treatment modalities for patients with maximal EOR are needed.

Carmustine wafer (CW) implantation intensifies local therapy. Double-blind placebo-controlled studies conducted before the temozolomide era demonstrated that CW implantation prolongs survival in newly diagnosed and recurrent malignant gliomas [4, 5]. Despite the lack of prospective data demonstrating an additive effect of CWs on radiation and temozolomide in newly diagnosed glioblastoma, a large French retrospective study demonstrated that CW implantation prolongs progression-free survival (PFS) in patients with total and subtotal resection [6]. Thus, this treatment is expected to be particularly effective in cases with a high EOR [7].

Vascular endothelial growth factor (VEGF) has angiogenic and vascular permeability-enhancing effects at sites of ischemia [8]. Bevacizumab is a humanized anti-VEGF antibody that normalizes blood vessels, decreases tissue interstitial pressure, and induces tissue reoxygenation by inhibiting abnormal angiogenesis and reducing vascular permeability [9, 10]. Synergistic effects of bevacizumab on other chemotherapeutic agents are expected through reoxygenation, and additive effects of bevacizumab in combination with several other agents have been examined [10]. Two placebo-controlled, double-blind, clinical trials were conducted to examine the effect of bevacizumab on temozolomide and radiotherapy in newly diagnosed glioblastoma [11, 12]. Both trials failed to demonstrate an OS benefit but showed an increase in PFS in the bevacizumab groups [11, 12]. A systematic review, however, reported that a combination of chemotherapeutic agents and bevacizumab had a modest effect on OS [13]. The additive effect of bevacizumab on CW implantation and temozolomide has not yet been prospectively examined. Instead of multiple combination treatments with carmustine, temozolomide, and bevacizumab, we reduced the number of cycles of maintenance treatment to avoid complications associated with the long-term administration of temozolomide and bevacizumab, such as myelodysplastic syndrome and acute myeloid leukemia after temozolomide [14]; and stroke [15], hypertension, and proteinuria [16] after bevacizumab.

This phase II study evaluated the efficacy and safety of CW implantation in combination with concomitant radiotherapy, temozolomide, and bevacizumab, and maintenance treatment limited to six cycles in patients with newly diagnosed glioblastoma who had no residual measurable disease after tumor resection according to the Response Assessment in Neuro-Oncology criteria (RANO) [17] in all registered patients.

## Methods

### Patients

We applied for a two-step registration. The inclusion criteria for the first registration were as follows: age 20–75 years, suspicion of supratentorial glioblastoma on preoperative gadolinium-enhanced T1-weighted magnetic resonance (MR) imaging (Gd-T1WI), and expectation for achieving an EOR ≥ 90% of the CE lesion. Patients were secondarily registered within 3–20 days after tumor resection. The inclusion criteria for the second registration were as follows: histological diagnosis of glioblastoma according to the fourth edition of the World Health Organization (WHO) Classification of Tumours of the Central Nervous System[18]; performance status (PS) of 0, 1, 2, or 3 on the Eastern Cooperative Oncology Group performance status (ECOG-PS) scale; and no measurable CE lesions based on RANO criteria [17] detected on Gd-T1WI within 72 h after tumor resection. The eligibility criteria for systemic conditions are provided in the Supplementary files.

### Treatment protocol

**Fig. 1:**
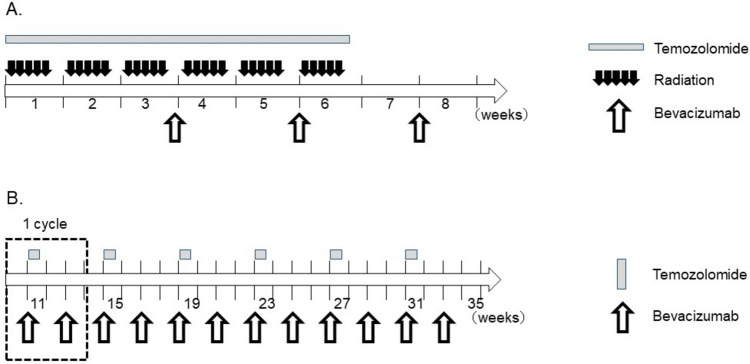
Treatment protocol of this study. **A** Concurrent radiation and chemotherapy phase. Within 21 days after maximal and safe tumor resection and implantation of carmustine wafers, patients received radiotherapy (60 Gy/30 fractions) as 3-dimensional conformal radiation therapy or intensity-modulated radiation therapy concomitantly with temozolomide (75 mg/m^2^, daily) from the first to last day of radiation therapy. Bevacizumab was also intravenously administered at a dose of 10 mg/kg, on day 1 of weeks 4, 6, and 8 after initiation of the radiation and temozolomide therapy. **B** Maintenance chemotherapy phase. Combination therapy with temozolomide (100–200 mg/m^2^ per day on days 1–5) and intravenous bevacizumab (10 mg/kg, on days 1 and 15 of each cycle) were started 4 weeks after ending the concomitant treatment and administered for 6 cycles (24 weeks)

Maximal and safe resection of the CE lesion on Gd-T1WI was performed. After confirming the pathological diagnosis of malignant glioma during surgery, one to eight CWs were implanted in the resection cavity. Within 21 days after tumor resection, patients received radiotherapy and temozolomide. Bevacizumab was also intravenously administered three times (Fig. 1A). In the maintenance chemotherapy phase, combination therapy with temozolomide and bevacizumab was started 4 weeks after ending the concomitant treatment and administered for six cycles (Fig. 1B).

### Patient evaluation and follow-up

The central histopathological diagnoses for all patients were reviewed according to the fourth edition of the WHO Classification of Tumours of the Central Nervous System [18] by consensus of two board-certified pathologists (H.S., H.T.).

Before the first and second registration and during protocol treatment and follow-up, patients underwent a physical examination, including subjective and objective symptoms, evaluation of Karnofsky Performance Status (KPS) and ECOG-PS, neurological examinations, blood cell counts, serum chemical examination, and urine examination. MR imaging was performed within 7 days before the first registration, within 72 h after tumor resection, and between the last day of concomitant radiation and chemotherapy and the first day of maintenance treatment. Thereafter, MR imaging was performed every 8 weeks.

### Statistical analysis

The primary endpoint was the 2-year OS rate in all patients with glioblastoma treated according to the protocol. OS is defined as the time from the second registration to death from any cause and was censored on the last day on which the patient was confirmed to be alive. Secondary endpoints were PFS, local PFS (LPFS), KPS deterioration-free survival time in all patients with glioblastoma treated according to protocol, and the incidence of adverse events (AE) and severe AEs in all registered patients. PFS was defined as the time from the second registration to disease progression or death from any cause and was censored the last day on which the patient was confirmed to be alive without any evidence of disease progression. Progression was defined according to the RANO criteria [17] (Supplementary file) and assessed by local neurosurgeons. The definitions of LPFS and KPS deterioration-free survival time are provided in the Supplementary file. AEs were evaluated according to the National Cancer Institute Common Terminology Criteria for Adverse Events, version 4.0.

We assumed an expected 2-year survival rate of 50% and a threshold 2-year survival rate of 35%, which were derived from patients who underwent gross total resection of the CE lesion and received concomitant radiation and temozolomide therapy with adjuvant temozolomide in the EORTC-NCIC study [19]. To obtain a power of 80% with a one-sided significance level of 5% for the 24-month registration and 36-month observation periods, 44 patients were required. Considering the number of patient withdrawals and exclusions at the second registration, the total target sample size was 55 patients. The Kaplan–Meier method was used to analyze the survival rate, and 90% confidence intervals (CIs) were calculated using the Greenwood formula. Because the number of patients was lower than initially predicted, we extended the registration period from 2 to 2.5 years and changed the significance level from 5 to 10%.

### DNA analysis

To elucidate the background of glioblastoma, *IDH1* and *IDH2* gene mutations; the *MGMT* gene promoter methylation status; and copy number alterations (CNAs) of *epidermal growth factor receptor* (*EGFR*), *cyclin-dependent kinase inhibitor* *2A* (*CDKN2A*), and *phosphatase and tensin homolog deleted from chromosome* *10* (*PTEN*) gene were evaluated as previously described (Supplementary file) [20–22].

## Results

### Patients

**Fig. 2:**
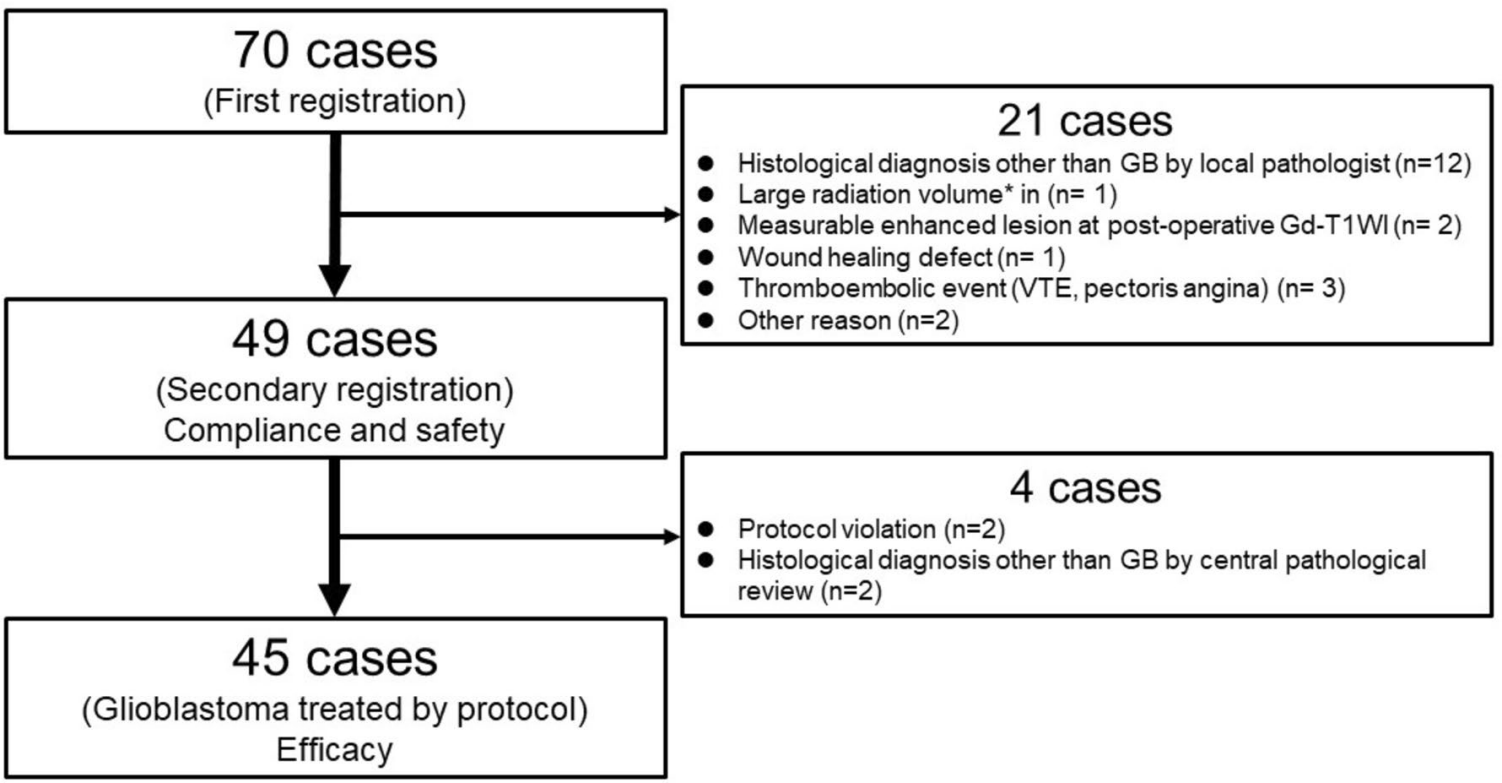
Patient flow in this study

Patient flow through the study is shown in Fig. 2. From October 2015 to April 2018, 70 patients from 17 institutes provided their consent to participate in the study, and the first registration was conducted. At the second registration, 21 patients were not eligible for registration for the reasons shown in Fig. 2. Thus, 49 cases were treated according to the protocol and analyzed for compliance and safety. Of these 49 cases, 4 were excluded from the analysis for efficacy; 2 patients received 12 cycles of maintenance temozolomide and bevacizumab, which was regarded as a protocol violation; and 2 patients were diagnosed with anaplastic ependymoma and anaplastic astrocytoma at the central pathological diagnosis. All 49 patients were of Asian ethnicity and Japanese nationality. The background of the 45 patients analyzed for efficacy is shown in Table 1.

**Table 1:** Patient demographics

	Number of patients (%)
Age (median) (mean)	33–75 (64.0) (59.7)
Sex	
Male	23 (51.1)
Female	22 (48.9)
ECOG PS at first registration	
0	9 (20.0)
1	17 (37.8)
2	9 (20.0)
3	10 (22.2)
KPS at first registration	
100	5 (11.1)
90	16 (35.6)
80	4 (8.9)
70	7 (15.6)
60	8 (17.8)
50	3 (6.7)
40	2 (4.4)

*ECOG PS*, European Clinical Oncology Group performance status; *KPS* Karnofsky performance status

### Protocol treatment compliance

Of the 49 cases, 16 (33%) completed the protocol treatment without any cessation or discontinuation of radiation therapy, temozolomide, or bevacizumab, and 43 (88%) cases completed 6 cycles of maintenance protocol treatment with cessation of chemotherapy or discontinuation of either radiation, temozolomide, or bevacizumab. Details of the protocol treatment compliance are provided in the Supplemental file.

### Primary and secondary outcome

**Fig. 3:**
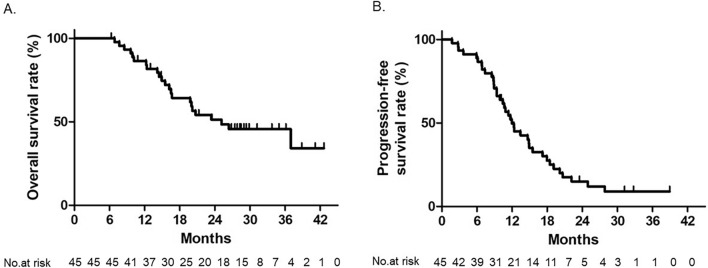
Overall survival (OS) (**A**) and progression-free survival (PFS) (**B**) rate in 45 glioblastoma patients who received protocol treatment. The 2-year OS rate was 51.3%. The median OS and PFS were 25 months and 11 months, respectively

Of the 45 glioblastoma patients treated according to protocol, 23 died during the study period. The estimated 2-year survival rate was 51.3% with an 80% CI of 40.8–60.9% (Fig. 3A). Median OS was 24.8 months (80% CI 19.7–36.4 months), with an OS rate of 34.2% at 45 months from the date of enrollment; 39 patients showed progression during the study period. The median PFS was 11.8 months (80% CI 10.5–13.3 months) (Fig. 3B), and the PFS rate at 39 months from the date of the definitive registration was 8.5%. Of the 39 cases with recurrence, 7 (18%) cases had distant recurrence and 32 (82%) had local recurrence. LPFS and KPS deterioration-free survival rates are shown in Supplementary Fig. 1.

### Adverse events

Table 2 provides a summary of the AEs observed in this study. All deaths were ruled out as causally related to protocol treatment during the study period.

**Table 2:** Summary of adverse effects (number of patients with adverse effects) in 49 registered patients

	All grades (%)*	Grade 3 or 4 (%)
Any adverse effects	44 (90)	26 (53)
Hematological adverse effects		
Any hematological adverse effects	33 (67)	15 (31)
Anemia	16 (33)	1 (2)
Lymphocyte count decreased	18 (37)	10 (20)
Neutrophil count decreased	17 (37)	9 (18)
Platelet count decreased	7 (14)	2 (4)
Non-hematological adverse effects		
Any non-hematological adverse effects	40 (82)	17 (35)
Fever	2 (4)	0
Wound dehiscence	1 (2)	0
Seizure	6 (12)	3 (6)
Hypertension	4 (8)	2 (2)
Proteinuria	8 (16)	3 (6)
Mucocutaneous bleeding	0	0
Apatite loss	7 (14)	1 (2)
Hyponatremia	5 (10)	0
Hyperkalemia	2 (4)	1 (2)
Allergic reaction	2 (4)	0
Infections and infestations	5 (10)	1 (2)
AST increased	8 (16)	1 (2)
ALT increased	5 (10)	0
Creatinine increased	2 (4)	0
Hydrocephalus	1 (2)	1 (2)
Cerebrovascular ischemia	6 (12)	2 (4)

*Assessed by Common Terminology Criteria for Adverse Effects version 4.0

*AST* aspartate aminotransferase, *ALT* alanine aminotransferase

Adverse effects known to occur with CWs and bevacizumab included proteinuria in 8 (16%), seizure in 6 (13%), cerebrovascular ischemia in 6 (12%), hypertension in 4 (8%), and wound dehiscence in 1 (2%). Cerebrovascular ischemia occurred between 8 and 25 months (median 14 months) after the second registration despite the short course of bevacizumab; cerebrovascular ischemia occurred in 5 patients after completion of the protocol therapy and in 1 patient during the maintenance phase.

### Survival by the MGMT gene promoter methylation status and CNA of EGFR, CDKN2A, and PTEN genes

The molecular profiles of our patients are shown in Supplementary Table 1. Median OS was not reached within the median follow-up period of 28.6 and 20.0 months in patients with methylated and unmethylated *MGMT* gene promoters, respectively (Supplementary Fig. 2). CNAs had no impact on the OS rate (data not shown).

## Discussion

In this multicenter phase II trial, Japanese patients with newly diagnosed glioblastoma who were candidates for complete resection (> 90%) of CE lesions were treated with maximal resection and CW implantation. Patients with no residual measurable CE lesion received concurrent treatment with radiation, temozolomide, and bevacizumab, and maintenance treatment was limited to six cycles of temozolomide and bevacizumab. The 2-year survival rate of 51.3% meets the prespecified criteria of a 2-year survival rate of > 50.0% in this study. This is the first prospective study demonstrating the result of maximal resection of newly diagnosed glioblastoma in Asian patients.

Compared to the subgroup analysis of patients with complete resection in the EORTC-NCIC study, which reported a 2-year survival rate of 38.4%, and in the EF-14 study, which demonstrated the effect of tumor treating fields with a median survival time of 22.6 months [23], this treatment was considered potentially beneficial. A similar regimen was examined in a retrospective study from a single Japanese institution [24]. Akiyama et al. reported a median OS of 24.2 months in patients treated with Stupp’s regimen plus CWs and bevacizumab [24]. Although maintenance therapy was continued until recurrence in their study, the patient background characteristics, including the EOR, were similar between our study and theirs (no measurable lesion vs 96%; mean age 59.7 years vs 62.2 years; and mean pretreatment KPS 76.6 vs 76.7), and the median OS was comparable. A study published in 2018, however, reported a significantly longer survival time in Asian glioblastoma patients than in Hispanic patients based on a large population [25]. This difference was explained by a difference in the proportion of cases with *EGFR* gene abnormalities or with *EGFR*, *CDKN2A*, and *PTEN* gene abnormalities [22, 26]. CNA analysis in the present study revealed that the CNA profiles of the *EGFR*, *CDKN2A*, and *PTEN* genes and the proportions of cases with CNAs for all three genes were similar to those of the Japanese cohort and significantly different from those of the TCGA cohort [22] (Supplementary Table 1). Therefore, because previous reports were limited to subgroup analysis, the results of this study should be compared to those of an Asian glioblastoma population with maximal resection [24, 27–34] (Supplementary Table 2). First, we reviewed previous reports in which the PFS and OS after maximal resection followed by Stupp’s regimen were reported based on more than 50 Asian patients [27–32]. Among these reports, the median OS exceeded 24 months in three studies [28–30]. In these studies, 52–58 patients were analyzed, and maximal resection was defined as 100% [29, 30] and > 99% [28] resection of CE lesions. In contrast, the median OS was 21.0 and 23.0 months in the large series from Korea [27] and China [32], respectively. Second, we reviewed the PFS and OS after maximal resection followed by Stupp’s regimen and CW implantation. The median OS in patients treated with CWs in combination with Stupp’s regimen was 22.3 months in a multicenter retrospective study [33] and 27.3 months in a postmarketing study that included patients under 70 years of age [34]. Based on those reports, adding the combination of CWs and bevacizumab to Stupp’s regimen or Stupp’s regimen and CW implantation could provide an additional benefit for OS. It is difficult to draw a definitive conclusion about the positive effects of adding CWs and bevacizumab, however, because of the large variability in this study (80% CI for median survival time: 19.7–36.4 months) and the unknown prognostic factors, such as *MGMT* promoter methylation status, KPS, and age, due to the subgroup analysis in previous reports.

A meta-analysis of clinical trials conducted in the United States and Europe found no effect of bevacizumab on survival in cases with methylated and unmethylated *MGMT* gene promoters [35]. Hata et al., however, demonstrated an additive effect of bevacizumab to Stupp’s regimen on OS in Japanese patients only with an unmethylated *MGMT* gene promoter [36]. In addition, Grossmann et al. reported an additive effect of CWs on OS in patients with a methylated *MGMT* gene promoter [37]. To explore whether the *MGMT* gene promoter methylation status affected the OS outcome of the combination of CW implantation and bevacizumab on glioblastoma, we compared our results to those of a multicenter retrospective study in Korean glioblastoma patients with total resection followed by Stupp’s regimen, in which the median OS was 28.6 months and 19.0 months in patients with methylated and unmethylated *MGMT* gene promoters, respectively [27]. While the median OS was comparable to that following Stupp’s regimen in patients with an unmethylated *MGMT* gene promoter (20.1 months vs 19.0 months), median OS in patients with a methylated *MGMT* gene promoter in the present study seems better (OS rate at 28.6 months: 74.0% vs 50%). Although these results were also obtained from the subgroup analysis and further analysis is required, CW implantation combined with bevacizumab might have an additive effect on Stupp’s regimen in patients with a methylated *MGMT* gene promoter. The addition of CW implantation to Stupp’s regimen was associated with longer PFS only in patients with subtotal or total resection [6] and improved local tumor control [38, 39]. A randomized control study demonstrated that the addition of bevacizumab to Stupp’s regimen was associated with longer PFS only in patients with tumor resection [6]. Based on these results, we expected that the addition of CWs and bevacizumab could lead to a significant improvement in PFS, and that the proportion of local recurrence would be decreased by maximum resection of gadolinium-enhanced lesions followed by CW implantation [33]. Only modest differences, however, were observed in the median PFS time and PFS rate at 2 years compared with previous reports (Supplementary Table 2) and 82% of the patients experienced local recurrence. We presumed that a short course of bevacizumab in the maintenance phase could be associated with a short median PFS because PFS time was prolonged when bevacizumab was continued until progression in clinical trials evaluating the addition of bevacizumab to Stupp’s regimen [6] and in a retrospective analysis of the addition of CW implantation and bevacizumab to Stupp’s regimen [24]. In this study, several factors may have contributed to the lack of a reduction in local recurrence. First, some reports demonstrated that resection must be performed beyond the gadolinium-enhanced lesion to reduce local recurrence [40, 41]. We did not collect data on the EOR of non-enhanced lesions in this study, but it is possible that the EOR was not sufficient to reduce local recurrence. Second, although a study by the French Neurosurgical Society demonstrated that CW implantation prolongs PFS in patients with subtotal and total resection of gadolinium-enhanced lesions based on a large number of patients, the effect of CWs on the pattern of recurrence remains unclear, especially in patients with total resection of gadolinium-enhanced lesions. Third, the effects of bevacizumab on the pattern of failure are also unclear. Although bevacizumab does not affect the pattern of recurrence [42, 43], its impact on the pattern of failure remains unknown [44].

In the present study, neutropenia and thrombocytopenia ≥ grade 3 were observed in 18% and 4% of patients, respectively. The incidence was comparable to that reported by the EORTC-NCIC (7% and 12%, respectively) and the JCOG 0911 (neutropenia: 16.2%) studies. The combination of CWs and bevacizumab could increase wound dehiscence. This complication was observed in only one patient (2%), comparable to the result of Stupp’s regimen (1.2–4.9%), Stupp’s regimen plus bevacizumab (2.6–6.9%), and Stupp’s regimen plus CW implantation (1.6–3.3%) [11, 12, 33, 34]. Limited cycles of maintenance treatment with bevacizumab and temozolomide could lead to a decrease in proteinuria, hyperextension, and mucocutaneous bleeding compared to previous reports [11, 12, 45] in which proteinuria (15.6–29.8%), hypertension (39.3–42.6%), and mucocutaneous bleeding (1.7–10.6%) were reported. Cerebrovascular ischemia, however, occurred more frequently (12%) than in previous reports reporting ischemic complications: 1.9% of cases from previous clinical trials with bevacizumab and other antiangiogenic agents [15] and 5.9–7.4% of cases in prospective clinical studies of Stupp’s regimen and bevacizumab [11, 45]. The combination of CWs and radiation, temozolomide, and bevacizumab itself could increase this complication by an unknown mechanism. Otherwise, considering that this complication occurred after completion of the maintenance phase in five of six cases, it might be due to salvage treatment.

The present study has some limitations. First, among the 70 patients initially enrolled, 21 (30%) did not proceed to the second registration. The most frequent reason was pathological diagnosis other than glioblastoma in 12 cases (17%), but we experienced 6 (8.5%) patients who did not proceed to second registration for reasons related to surgery, including delayed wound healing, pulmonary embolus, and residual lesion in 2 cases (2.9%). Second, information on salvage treatment is lacking. The protocol was limited to six courses of maintenance temozolomide and bevacizumab. In contrast to previous clinical trials of bevacizumab for newly diagnosed glioblastoma in which bevacizumab was administered until progression, limited cycles of bevacizumab in this study may lead to the early detection of recurrence. In addition, 88% of the patients were able to complete at least part of the planned treatment. These results suggest that the patients might have a chance to receive a rechallenge of temozolomide and bevacizumab at recurrence, and the effect of salvage treatment could significantly affect the outcome. An analysis of salvage treatment might have clarified the mechanism underlying the excellent OS. Third, this study included patients without measurable enhanced lesions on postoperative MR images. Quantitative estimation of the CE lesion's EOR or residual volume is required for comparison with other studies, as shown in Supplementary Table 2.

## Conclusion

This phase II study demonstrated the efficacy and safety of CW implantation followed by concomitant radiation, temozolomide, and bevacizumab, and six cycles of temozolomide and bevacizumab in patients with newly diagnosed glioblastoma. Although the hematological AE was identical to that of Stupp’s regimen, strokes frequently occurred. This is the first prospective study demonstrating the result of maximal resection of newly diagnosed glioblastoma in Asian patients. The addition of CW and bevacizumab to Stupp’s regimen might provide an additional benefit for survival in Japanese glioblastoma patients with maximal resection.

## Supplementary Information

Below is the link to the electronic supplementary material.

Supplementary file1 (DOCX 27 KB)

Supplementary file2 (DOCX 130 KB)

Supplementary file3 (DOCX 20 KB)

## Data Availability

The datasets used in the present study are available from the corresponding author upon request. All data generated or analyzed in this study are included in this published article. M. Kanamori had full access to all the data in the study and takes responsibility for the integrity of the data and accuracy of the data analysis.
