# Supplementary material for: Efficacy and safety of carmustine wafers, followed by radiation, temozolomide, and bevacizumab therapy, for newly diagnosed glioblastoma with maximal resection
Source: Int J Clin Oncol. 2024 Nov 11;30(1):51–61. doi: 10.1007/s10147-024-02650-9 (PMC11700082; doi:10.1007/s10147-024-02650-9)
Supplement: Supplementary file 2 — Supplementary file2 (DOCX 130 KB) [file 10147_2024_2650_MOESM2_ESM.docx]

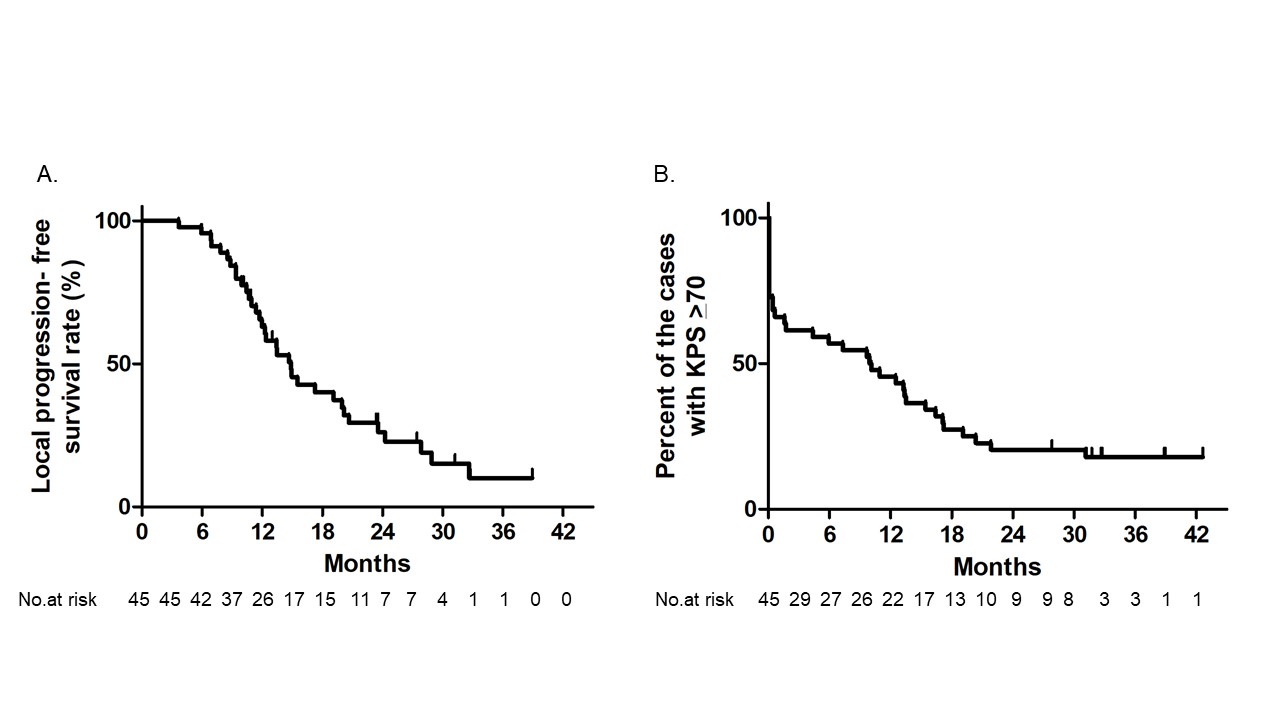
Supplemental Fig. 1 Local progression-free survival (LPFS) and Karnofsky performance status (KPS) deterioration-free survival rate in 45 cases. The median LPFS was 14.7 months (80% CI: 12.2–17.0 months) (Supplemental Fig. 1A), and the LPFS rate at 39 months from the date of the definitive registration was 10.1%. The median time that KPS was maintained ≥70 was 10.8 months (80% CI: 5.8–13.3 months) (Supplemental Fig. 1B).


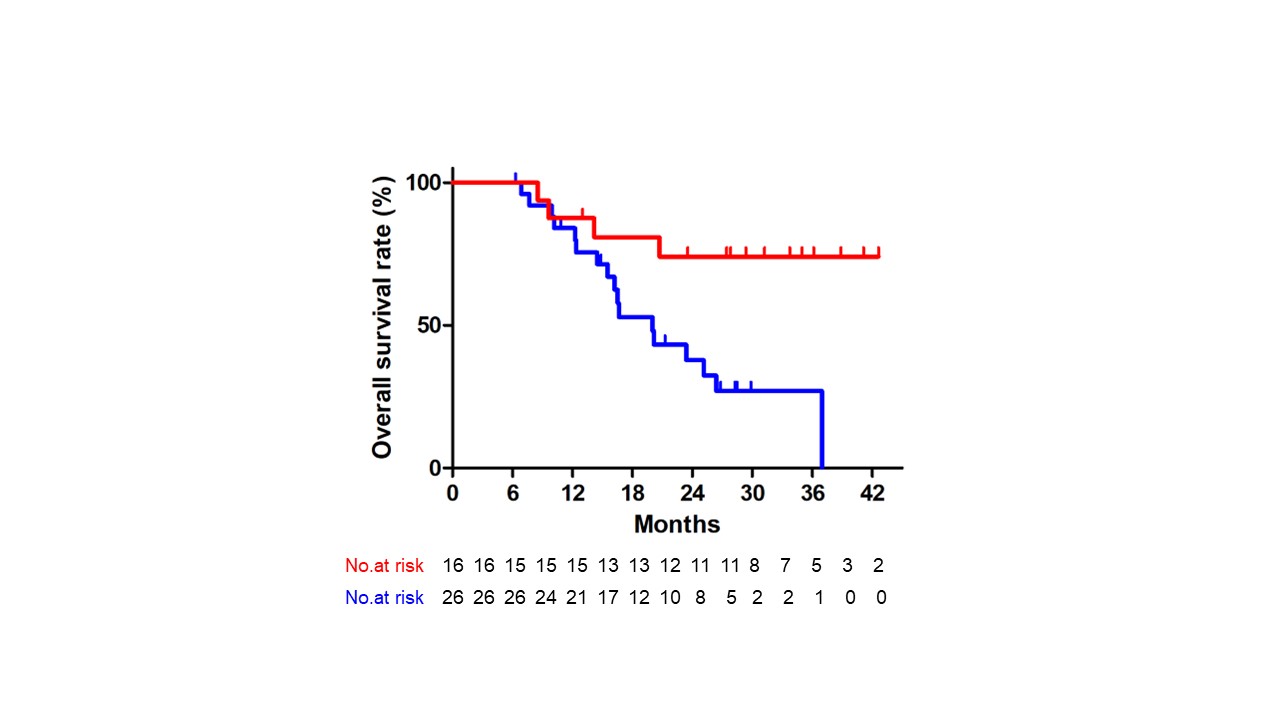


Supplemental Fig. 2 Overall survival rate in patients with methylated (red) and unmethylated (blue) *MGMT* gene promoters. Median OS was not reached with a median follow-up of 28.6 months and 20.0 months in patients with methylated and unmethylated *MGMT* gene promoters, respectively.
