# Supplementary material for: Efficacy and safety of carmustine wafers, followed by radiation, temozolomide, and bevacizumab therapy, for newly diagnosed glioblastoma with maximal resection
Source: Int J Clin Oncol. 2024 Nov 11;30(1):51–61. doi: 10.1007/s10147-024-02650-9 (PMC11700082; doi:10.1007/s10147-024-02650-9)
Supplement: Supplementary file 3 — Supplementary file3 (DOCX 20 KB) [file 10147_2024_2650_MOESM3_ESM.docx]

Supplementary file

**Material and Methods**

*Patients*

The eligibility criteria for systemic condition were as follows: neutrophil count ≥1500/mm3, platelet count ≥100,000/mm3, hemoglobin ≥8.0g/dl, aspartate transaminase ≤120 IU/L alanine transaminase ≤120 IU/L, creatinine ≤1.5 mg/dl, urine protein ≤1+, and prothrombin time-international normalized ratio (PT-INR) ≤1.5. Patients with postoperative symptomatic intracranial hemorrhage, angina pectoris, thromboembolism, gastrointestinal perforation, and intracerebral abscess were excluded.

*Statistical analysis*

Progression was defined according to the RANO criteria. None of the patients had measurable CE lesions at the second registration. Therefore, clear progression of non-measurable CE lesions or a significant increase in hyperintense lesions on T2WI or FLAIR compared with the images at baseline or best response on stable or increasing doses of corticosteroids; any newly developing lesions; clinical deterioration corresponding to a decrease in KPS ≥20 attributable to the tumor; increase in the corticosteroid dose;

LPFS was defined as the number of months from the second registration to local progression. Local progression was defined as the development of a recurrent lesion within 2.0 cm from the resection cavity, as previously reported [1]. KPS deterioration-free survival was defined from the second registration to the time of deterioration to KPS <70.

*DNA analysis*

Written informed consent was obtained from all study participants. Molecular analysis was approved by the Institutional Review Board of Tohoku University Hospital (2015-2-126-1, 2023-1-971). DNA was extracted using the QIAamp DNA Mini Kit (Qiagen, Valencia, CA, US) after sodium bisulfate modification with the CpG genome DNA modification kit (Qiagen). For estimation of the mutation in the *IDH1* and *IDH2* genes, exon 4 of the *IDH1* gene, including codon 132, and exon 4 of the *IDH2* gene, including codon 172, were amplified from 100 ng genomic DNA by polymerase chain reaction using primers [2]. After purification of the DNA fragments, sequence reactions were carried out using the GenomeLab DTCS quick-start kit (Beckman Coulter, Fullerton, CA, USA). The reactions were carried out in an automated DNA analyzer (CEQ 8000; Beckman Coulter). Genomic DNA (2 µg) was treated with sodium bisulfite using the CpG genome DNA modification kit (Qiagen). Modified DNA was amplified by polymerase chain reaction using primer sequences specific to unmethylated or methylated promoter sequences [3]. DNA fragments were separated on 4% agarose gels and visualized with ethidium bromide. CNAs of *EGFR*, *CDKN2A*, and *PTEN* genes were analyzed by multiplex Ligation-dependent Probe Amplification (MLPA) using a SALSA MLPA kit (P-105, version D3). Amplification products were separated on an ABI-3730XL Genetic Analyzer (Applied Biosystems, Foster City, CA). Quantitative data at each target region were analyzed using the Coffalyser software, version 220513.1739 (MRC Holland; www.coffalyser.net), where relative probe values of probe-amplified products are compared with normal control DNA from white blood cells of healthy individuals. The CNA category was classified by the following thresholds: homozygous deletion (≤0.4), hemizygous deletion (0.4–0.7), gain (1.3–2.0), and amplification (2.0) [4].

**Results**

*Protocol treatment compliance*

We examined protocol compliance in 49 patients. In the concurrent phase, radiation therapy, temozolomide, and bevacizumab were discontinued in 1 (2%), 4 (8%), and 1 (2%) patients. Between the concurrent phase and maintenance phase, 4 patients discontinued the protocol treatment due to recurrence, and 1 discontinued for an unknown reason. In the maintenance phase, temozolomide and bevacizumab were discontinued in 4 (8%) and 7 (14%) cases.

Reference

1. Hochberg FH, Pruitt A. Assumptions in the radiotherapy of glioblastoma. Neurology. 1980;30(9):907-911. doi: 10.1212/wnl.30.9.907. PMID: 6252514.
2. Sonoda Y, Kumabe T, Nakamura T, et al. Analysis of IDH1 and IDH2 mutations in Japanese glioma patients. Cancer Sci. 2009;100(10):1996-1998. doi: 10.1111/j.1349-7006.2009.01270.x. PMID: 19765000.
3. Sonoda Y, Kumabe T, Watanabe M, et al. Long-term survivors of glioblastoma: clinical features and molecular analysis. Acta Neurochir (Wien). 2009;151(11):1349-1358. doi: 10.1007/s00701-009-0387-1. PMID: 19730774.
4. Umehara T, Arita H, Yoshioka E, et al. Distribution differences in prognostic copy number alteration profiles in IDH-wild-type glioblastoma cause survival discrepancies across cohorts. Acta Neuropathol Commun. 2019;7(1):99. doi: 10.1186/s40478-019-0749-8. Erratum in: Acta Neuropathol Commun. 2019 Aug 14;7(1):131. PMID: 31215469; PMCID: PMC6580599.
