# Supplementary material for: Efficacy and safety of carmustine wafers, followed by radiation, temozolomide, and bevacizumab therapy, for newly diagnosed glioblastoma with maximal resection
Source: Int J Clin Oncol. 2024 Nov 11;30(1):51–61. doi: 10.1007/s10147-024-02650-9 (PMC11700082; doi:10.1007/s10147-024-02650-9)
Supplement: Supplementary file 3 [file 10147_2024_2650_MOESM1_ESM.docx]

Supplementary Table 1. Molecular backgrounds of the patients in this study

		Number of patients (%)	Japanese cohort (%) (n= 212)[26)]	TCGA cohort (%) (n=359)[26)]
IDH gene mutation	Wild-type	44 (97.8)	212 (100)	359 (100)
	Mutant	1 (2.2)	0	0
MGMT gene promoter	Methylated	16 (38.1)	98 (46.2)	110 (41.5)
	Unmethylated	26 (61.9)	114 (53.8)	155 (58.5)
EGFR	Amplification	15 (37.5)	54 (25.5)	181 (50.4)
	Gain	7 (17.5)	65 (30.7)	159 (44.3)
	No gain/amplification	18 (45.0)	93 (43.8)	19 (5.3)
CDKN2A	Homozygous deletion	13 (32.5)	79 (37.3)	210 (58.5)
	Hemizynous deletion	8 (20.0)	53 (25.0)	61 (17.0)
	Retain	19 (47.5)	80 (37.5)	88 (24.5)
PTEN	Homozygous deletion	2 (5.0)	4 (1.9)	41 (11.6)
	Hemizygous deletion	18 (45.0)	90 (42.5)	300 (83.6)
	Retain	20 (50.0)	118 (55.6)	18 (4.8)
Triple CNA	Triple	12 (30.0)	51 (24.1)	253 (70.5)
	Non-triple	28 (70.0)	161 (75.9)	106 (29.5)

Abbreviations: IDH, isocitrate dehydrogenase; MGMT, O6 -methylguanine-DNA methyltransferase; EGFR, epidermal growth factor receptor; CDKN2A, cyclin-dependent kinase inhibitor 2A; PTEN, phosphatase and tensin homolog deleted from chromosome 10; CNA, copy number alterations

Supplementary Table 2, Progression-free survival and overall survival in Asian glioblastoma patients after maximal resection.

Authors	Number of cases	Age (year-old)	Type of study	Treatments	KPS at diagnosis	Mutation in the *IDH* gene (%)	Cycles of adjuvant TMZ	PFS at 2 year	median PFS	OS at 2 year	median OS	Extent of resection of CE
Kim et al [27].	388	N.D.	Retrospective, multicenter	RT/TMZ	N.D.	N.D.	0-15 (median 6)		10.9		21.0	>99%
Joo et al [28].	57	N.D.	Prospective, multicenter	RT/TMZ	≥ 60	N.D.	1-15 (median 6)	48.7		50.1	25.0	>99%
Woo et al [29]	52	N.D.	Retrospective multicenter	RT/TMZ	N.D.	N.D.	N.D.		N.D.		24.6	100%
Ishikawa et at [30]	58	N.D.	Retrospective single center	RT/TMZ	N.D.	N.D.	N.D.		11.2		25.6	100%
Ahn et et al [31]	91	N.D.	Retrospective single center	RT/TMZ	N.D.	N.D.	N.D.		ND		20.0[a]<br>21.0[b]	>90%
Jiang et al [32]	264	N.D.	Retrospective single center	RT/TMZ	N.D.	N.D.	N.D.		11.0		23.0	100%
Sonoda et al [33].	69	N.D.	Retrospective, multicenter	RT/TMZ/ Carmustine wafers	N.D.	N.D.	N.D.		10.9		22.3	100%
Iuchi et al [34].	101	≤70	Post-marketing surveillance	RT/TMZ/ Carmustine wafers	N.D.	N.D.	N.D.		ND	57.1	27.4	>95%
Akiyama et al [24].	25	61.1. (mean)	Retrospective, single-center	RT/TMZ	N.D.	N.D.	Until PD		7.3		15.3	93%
	29	62.2 (mean)		RT/TMZ/ Carmustine wafers/BEV	N.D.	N.D.	Until PD		16.8		24.2	96%
Present study	45	59.7 (mean)	Prospective, multi-center	RT/TMZ/ Carmustine wafers/BEV	KPS ≥60: 89%	2.2	0-6 (median 6)	49.6	11.8	51.3	24.8	NMR
	36	≤70					0-6 (median 6)			58.2	37.0	NMR

Abbreviations: ND, not described; RT, radiation to local site; TMZ, temozolomide; BEV, bevacizumab; KPS, Karnofsky Performance Status; PFS, progression-free survival; OS, overall survival, CE, contrast-enhanced lesion; NMR, no measurable residual lesion
